# Progression of Mitral Regurgitation in Rheumatic Valve Disease: Role of Left Atrial Remodeling

**DOI:** 10.3389/fcvm.2022.862382

**Published:** 2022-03-11

**Authors:** Nayana F. A. Gomes, Vicente Rezende Silva, Robert A. Levine, William A. M. Esteves, Marildes Luiza de Castro, Livia S. A. Passos, Jacob P. Dal-Bianco, Alexandre Negrão Pantaleão, Jose Luiz Padilha da Silva, Timothy C. Tan, Walderez O. Dutra, Elena Aikawa, Judy Hung, Maria Carmo P. Nunes

**Affiliations:** ^1^School of Medicine, Hospital das Clínicas, Federal University of Minas Gerais, Belo Horizonte, Brazil; ^2^Cardiac Ultrasound Lab, Harvard Medical School, Massachusetts General Hospital, Boston, MA, United States; ^3^The Center for Excellence in Vascular Biology, Cardiovascular Medicine, Brigham and Women's Hospital, Harvard Medical School, Boston, MA, United States; ^4^Department of Statistics, Federal University of Paraná, Curitiba, Brazil; ^5^Department of Cardiology, Blacktown Hospital, University of Western Sydney, Penrith, NSW, Australia; ^6^Department of Morphology, Institute of Biological Sciences, Federal University of Minas Gerais, and National Institutes for Science and Technology, Belo Horizonte, Brazil

**Keywords:** progression, atrial fibrillation, mitral stenosis, left atrial, mitral regurgitation, rheumatic heart disease

## Abstract

**Introduction:**

Mitral regurgitation (MR) is the most common valve abnormality in rheumatic heart disease (RHD) often associated with stenosis. Although the mechanism by which MR develops in RHD is primary, longstanding volume overload with left atrial (LA) remodeling may trigger the development of secondary MR, which can impact on the overall progression of MR. This study is aimed to assess the incidence and predictors of MR progression in patients with RHD.

**Methods:**

Consecutive RHD patients with non-severe MR associated with any degree of mitral stenosis were selected. The primary endpoint was a progression of MR, which was defined as an increase of one grade in MR severity from baseline to the last follow-up echocardiogram. The risk of MR progression was estimated accounting for competing risks.

**Results:**

The study included 539 patients, age of 46.2 ± 12 years and 83% were women. At a mean follow-up time of 4.2 years (interquartile range [IQR]: 1.2–6.9 years), 54 patients (10%) displayed MR progression with an overall incidence of 2.4 per 100 patient-years. Predictors of MR progression by the Cox model were age (adjusted hazard ratio [HR] 1.541, 95% CI 1.222–1.944), and LA volume (HR 1.137, 95% CI 1.054–1.226). By considering competing risk analysis, the direction of the association was similar for the rate (Cox model) and incidence (Fine-Gray model) of MR progression. In the model with LA volume, atrial fibrillation (AF) was no longer a predictor of MR progression. In the subgroup of patients in sinus rhythm, 59 had an onset of AF during follow-up, which was associated with progression of MR (HR 2.682; 95% CI 1.133–6.350).

**Conclusions:**

In RHD patients with a full spectrum of MR severity, progression of MR occurs over time is predicted by age and LA volume. LA enlargement may play a role in the link between primary MR and secondary MR in patients with RHD.

## Introduction

Rheumatic heart disease (RHD) remains a serious global health concern as the leading cause of cardiovascular death in children and young adults (1, 2). The prevalence of RHD has been rising steadily since 1990, reaching 40.5 million in 2019, and accounting for 306,000 deaths annually as a consequence of severe valvular disease (3). Mitral regurgitation (MR) is the most common valvular abnormality at the early RHD stages, usually associated with ongoing inflammatory rheumatic activity in children (4–7). This pure MR may resolve with effective treatment of the acute carditis and continued prophylactic therapy. In the late time course, MR is often associated with stenosis owing to intrinsic valvular lesions that include fibrosis with retracted leaflets, restricted mobility, and commissural fusion (8).

Although the mechanism by which MR develops in RHD is primarily related to the structural impairment of the mitral valve (MV) apparatus (8), longstanding volume overload with left-sided chamber alterations may trigger the development of secondary MR (9). Moreover, in the presence of atrial fibrillation (AF), which often occurs in patients with rheumatic MV disease, MR may also arise as a consequence of left atrial (LA) enlargement and mitral annular dilatation. Functional MR in patients with AF has been increasingly recognized (10–12). However, whether mitral annular dilatation causes MR in patients without left ventricular dysfunction remains controversial.

There is a growing awareness that MR continues to progress over time as the increased volume load on the left ventricle and LA results in geometric changes that lead to a further increase in the severity of MR (13, 14). Additionally, the most common pattern of MV pathology in middle-aged adults with RHD is mixed MV disease, which begets LA enlargement (8, 15). Taken together, both primary MR and secondary MR may coexist in the setting of RHD, which may have an impact on MR progression. However, because of the paucity of data available on the progression of rheumatic MR, the underlying mechanisms are not certain.

Previous studies addressing the progression of MR in adult patients with RHD have focused mainly on MR following valvuloplasty (16–20). In this context, the progression and prognosis are variables depending on the mechanism by which MR develops. We previously showed that MR originated at the site of commissural split or at the central orifice of the valve and remains stable over time. On the other hand, MR due to leaflet tearing at central scallop location or subvalvular damage results in severe adverse hemodynamics that require immediate surgery (20). However, there is a lack of studies on the natural history of rheumatic MR without intervention, as it requires large cohorts of patients with repeated echocardiograms and long-term follow-up. To fill these gaps of knowledge, we sought to investigate the incidence and predictors of MR progression in a substantial population of patients with RHD.

## Methods

### Study Population

**Figure 1:**
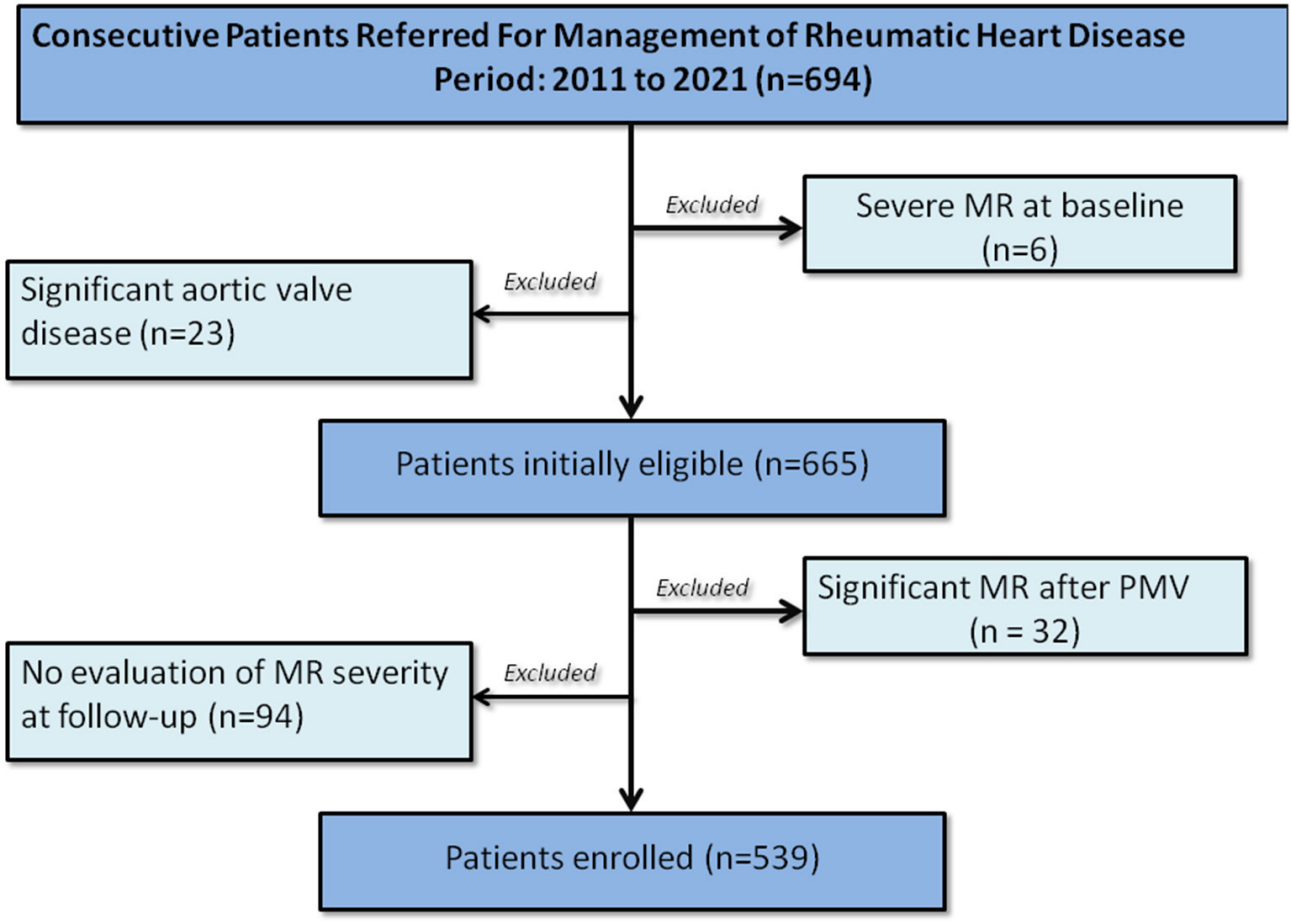
Study population.

Patients were recruited prospectively from a tertiary center for heart valve disease among those routinely referred for management of RHD from 2011 to 2021. Patients with rheumatic MV disease with trivial, mild, or moderate MR associated with any degree of mitral stenosis based on the presence of typical rheumatic features by echocardiography criteria (21) were initially eligible for the study (Study flow is shown in Figure 1). Exclusion criteria included severe MR at baseline or following percutaneous mitral valvuloplasty, associated significant aortic valve disease, and no echocardiographic assessment of MR at last follow-up. Among 694 patients initially eligible for the study, 539 fulfilled the inclusion criteria and were enrolled.

Information on demographic data, functional capacity, right-sided heart failure, and current medications was obtained at baseline. AF was diagnosed based on a history of permanent AF, supported by a past 12-lead ECG. A diagnosis of new-onset AF in patients with sinus rhythm at the time of enrollment in the study was confirmed by a 12-lead ECG. All patients gave written informed consent, and the study protocol was approved by the UFMG institutional ethics committee.

### Echocardiography

Comprehensive two-dimensional (2D) and Doppler echocardiographic examinations were performed in all patients at baseline and at follow-up using commercially available echocardiography machines. Measures of left ventricular dimensions and function were assessed as recommended (22). MR was graded as none/trace, mild, moderate, or severe by using an integrative approach (23). Parameters used to grade MR included the *vena contracta* width, regurgitant volume, and effective orifice area, qualitative assessment of the color flow jet, and, when available, the pulmonary venous flow signal. MV area was measured using direct planimetry. Peak and mean transmitral diastolic pressure gradients were measured from Doppler profiles recorded in the apical four-chamber view. The presence and severity of tricuspid regurgitation and systolic pulmonary artery pressure were evaluated according to the guidelines (20). LA volume was assessed by the biplane area-length method from apical 2- and 4-chamber views.

### Definition of MR Progression

Progression of MR was defined as an increase of one grade in MR severity from baseline to the last follow-up echocardiogram. Patients in whom MR did not progress but died or underwent MV replacement were censored at the time of the events and also analyzed considering these events as competing risks (24). Patients who underwent percutaneous mitral valvuloplasty were censored at the time of the procedure and post-procedural MR was not considered as progression.

### Statistical Analysis

Categorical variables were expressed as numbers and percentages and were compared by using chi-square or Fisher exact tests as appropriate. Continuous data were expressed as mean ± SD and were compared by using unpaired Student's t-test or Mann-Whitney test as appropriate.

The incidence rate of MR progression was calculated by dividing the number of progression by the person-years of follow-up calculated from the baseline until either the date of death or MV replacement or last follow-up echocardiogram.

Predictors of MR progression were assessed using two regression models. The first was the Cox proportional hazards model in which patients were censored at the time of death or MV replacement if it was not preceded by MR progression. The second model was the Fine-Gray competing risk model in which MR progression was the primary event and death or MV replacement was the competing risk (24) that may prevent progression of the valve regurgitation. The estimated regression coefficients for each variable were compared between the two models to assess differences in the direction of their association with the rate of MR progression (derived from the Cox model) vs. its incidence (derived from the Fine-Gray model) (24). Schoenfeld residuals were used to check the proportional hazards assumption.

Long-term MR progression according to cardiac rhythm was estimated by the Kaplan-Meier method and compared by the log-rank test. Statistical analysis was performed using the Statistical Package for Social Sciences for Windows, version 22.0 (SPSS Inc., Chicago, IL, USA) and R for Statistical Computing version 3.6.3 (R Foundation, Vienna, Austria).

## Results

### Patient Characteristics

Our final cohort consisted of 539 patients, age of 46.2 ± 12 years, and 454 patients were women (83%). Baseline demographic and clinical characteristics according to MR progression are summarized in Table 1. At baseline, trivial MR was detected in 80 patients (15%), mild MR in 416 (77%), and moderate in 43 (8%). Most of the patients were in the New York Heart Association (NYHA) classes I and II (64%), whereas 194 (36%) patients were in NYHA classes III and IV at presentation. One hundred and sixteen patients (22%) were presented with right-sided heart failure. The medications most frequently used were beta-blockers (74% of cases) followed by diuretics (69% of cases).

**Table 1:** Demographic and clinical characteristics of the study population stratified by mitral regurgitation (MR) progression.

Clinical data^*^	**No progression (*n* = 485)**	**MR progression (*n* = 54)**	***p* value**
Age (years)	45.7 ± 12.1	50.0 ± 13.1	**0.016**
Female gender (%)	400 (83)	47 (87)	0.398
NYHA class III-IV (n/%)	179 (37)	19 (36)	0.919
Right-sided heart failure	106 (22)	14 (27)	0.399
Atrial fibrillation (n/%)	149 (31)	24 (44)	**0.039**
Previous valvuloplasty^†^	170 (35)	15 (28)	0.293
Ischemic cerebrovascular events^‡^	97 (20)	6 (11)	0.131
Diuretics use	339 (70)	39 (75)	0.439
Anticoagulation therapy	90 (32)	23 (36)	0.535
Heart rate (bpm)	70.1 ± 13.8	71.1 ± 12.7	0.576
Systolic blood pressure (mmHg)	117.8 ± 15.8	115.5 ± 14.5	0.332
Diastolic blood pressure (mmHg)	75.5 ± 10.9	74.7 ± 10.7	0.637

* *Data are expressed as the mean value ± SD, or absolute numbers (percentage)*.

† *Surgical commissurotomy or percutaneous valvuloplasty*.

‡ *Stroke or transient ischemic attack at baseline*.

*Bold numbers mean a p-value < 0.05%*.

In the overall population, the left atrium was severely dilated, with a mean volume of 54 ml/m^2^ in the patients in sinus rhythm compared with 74 ml/m^2^ in AF (*p* < 0.001). One hundred and thirty-five patients (25%) had a history of hypertension and 3% of diabetes. The majority of patients had no comorbidities.

Regarding baseline echocardiographic characteristics, patients who progressed had larger left ventricular chamber dimensions, LA volume, and lower ejection fraction. Of note, the severity of the associated mitral stenosis was similar between the patients with a mean valve area of 1.1 cm^2^ in those patients who progressed or did not. Baseline echocardiographic features according to MR progression are summarized in Table 2.

**Table 2:** Baseline echocardiographic characteristics of the study population stratified by MR progression.

**Echocardiographic data**	**No progression (*n* = 485)**	**MR progression (*n* = 54)**	***p* value**
LVDd (mm)	48.4 ± 6.0	50.5 ± 6.7	**0.017**
LVSd (mm)	31.6 ± 5.2	33.7 ± 6.7	**0.005**
LVEF (%)	58.5 ± 6.8	55.7 ± 6.9	**0.009**
LAV index (mL/m^2^)	59.6 ± 23.9	67.9 ± 32.3	**0.027**
RA area (cm^2^)	17.5 ± 6.9	17.0 ± 5.4	0.620
Peak gradient (mmHg)	18.3 ± 7.2	16.6 ± 6.0	0.083
Mean gradient (mmHg)	10.1 ± 4.9	9.3 ± 4.0	0.227
Mitral valve area (cm^2^)^*^	1.14 ± 0.40	1.14 ± 0.36	0.996
SPAP (mmHg)	44.7 ± 17.0	40.3 ± 11.3	**0.025**
Systolic annular velocity (cm/s)^†^	10.5 ± 2.2	9.9 ± 2.1	**0.048**
Right ventricular FAC (%)	46.2 ± 10.1	48.7 ± 11.0	0.119
Moderate or severe TR (n/%)	77 (16)	6 (11)	0.384
C_n_ (mL/mmHg)	5.1 ± 1.9	5.6 ± 1.8	0.089

*Data are expressed as the mean value ± SD, or absolute numbers (percentage)*.

* *Mitral valve area by planimetry*.

† *Peak systolic velocity at the tricuspid annulus*.

*C_n_, net atrioventricular compliance; LA, left atrium; LAV, left atrial volume; LVDd, left ventricular end-diastolic diameter; LVEF, left ventricular ejection fraction; LVSd= left ventricular end-systolic diameter; MR, mitral regurgitation; RA, right atrium; FAC, fractional area change; SPAP, systolic pulmonary artery pressure; TR, tricuspid regurgitation*.

*Bold numbers mean a p-value < 0.05%*.

At a mean follow-up time of 4.2 years (interquartile range [IQR]: 1.2–6.9 years), 54 patients (10%) displayed MR progression. The majority of the patients progressed from mild-to-moderate MR (*n* = 42, 77.8%), mild-to-severe (*n* = 6, 11.1%), trivial MR-to-moderate (*n* = 3, 5.5%), and the other 3 patients (5.5%) from moderate-to-severe MR. Patients who progressed MR were older compared with those who did not progress. Permanent AF at baseline was found in 173 patients, more frequent in patients who had MR progression.

### Predictors of MR Progression

**Figure 2:**
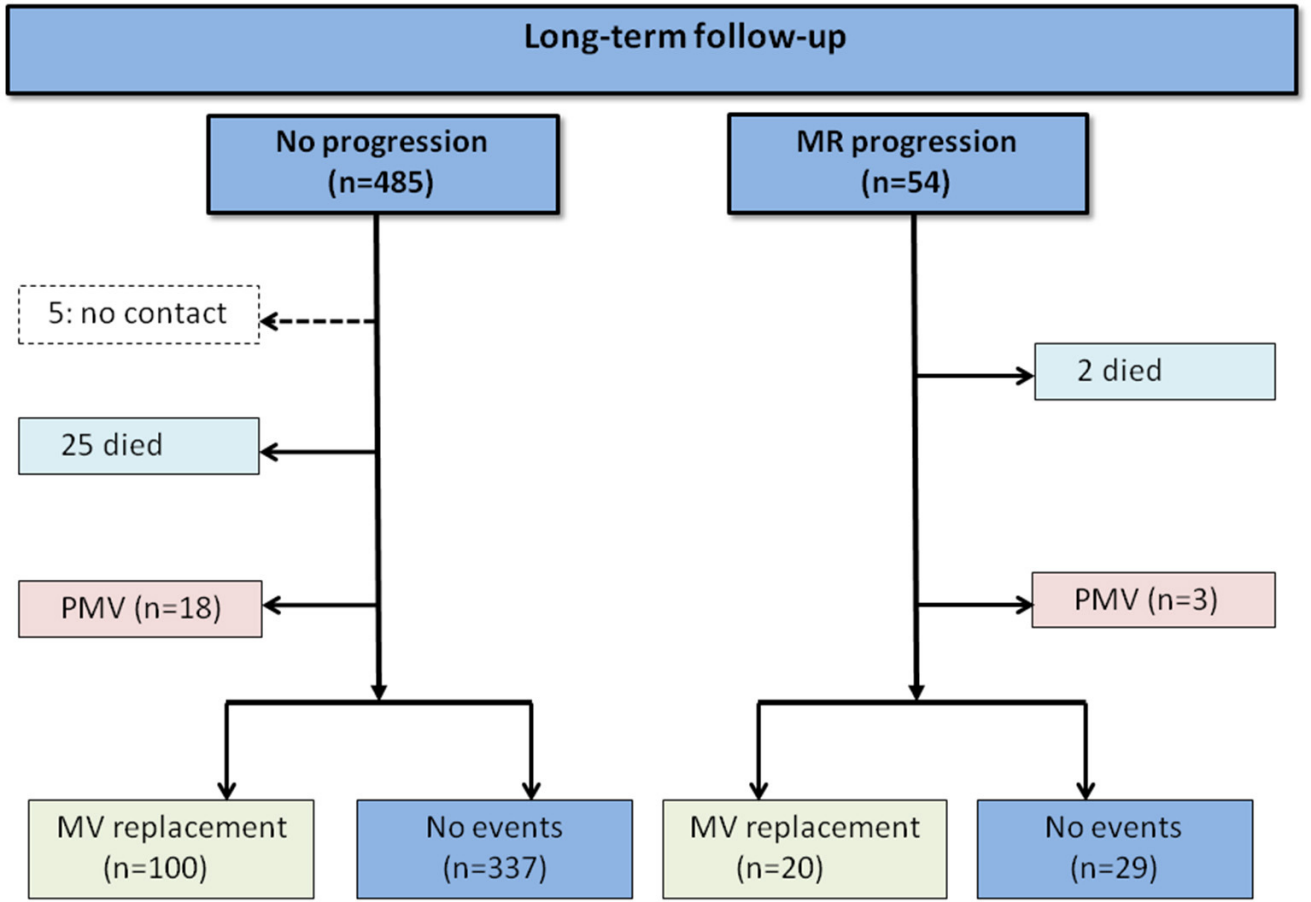
Long-term follow-up of patients with rheumatic heart disease (RHD).

The overall incidence of MR progression was 2.4 per 100 patient-years. During the follow-up, 120 patients underwent cardiac surgery for MV replacement and 27 patients died, being 23 cardiovascular-related and four non-cardiovascular-related deaths (Figure 2). In the Cox proportional hazard regression model, older age, the presence of AF, and larger LA volume were univariately associated with MR progression. Interestingly, prior mitral valvuloplasty, i.e., either percutaneous or surgical intervention, was inversely associated with MR progression. The severity of tricuspid regurgitation was not associated with MR progression (Table 3).

**Table 3:** Clinical and echocardiographic characteristics associated with MR progression in patients with RHD: Cox regression model.

**At baseline**	**Unadjusted**		**Multivariable model**		**Final model**	
	**HR (95% CI)**	***P* value**	**HR (95% CI)**	***P* value**	**HR (95% CI)**	***P* value**
Age^*^	1.563 (1.245–1.962)	0.000	1.486 (1.156–1.909)	0.002	1.541 (1.222 - 1.944)	**0.000**
Permanent AF	2.527 (1.467–4.354)	0.001	1.555 (0.781–3.095)	0.209		
LA volume index^*^	1.152 (1.069–1.241)	0.000	1.108 (1.014–1.211)	0.023	1.137 (1.054 - 1.226)	**0.001**
Prior PMV	0.453 (0.230–0.895)	0.023	0.493 (0.245–0.992)	0.047	0.479 (0.239 - 0.961)	**0.038**
Mild TR^†^	0.980 (0.291–3.302)	0.974	0.803 (0.235–2.752)	0.728		
Moderate TR	1.356 (0.310–5.938)	0.686	0.764 (0.160–3.647)	0.736		
Severe TR	0.465 (0.047–4.602)	0.512	0.253 (0.025–2.609)	0.249		

* *Hazard ratio: x10*.

† *Reference category was absence of tricuspid regurgitation*.

*LA, left atrium; AF, atrial fibrillation; PMV, percutaneous mitral valvuloplasty; TR, tricuspid regurgitation*.

*Bold numbers mean a p-value < 0.05%*.

**Figure 3:**
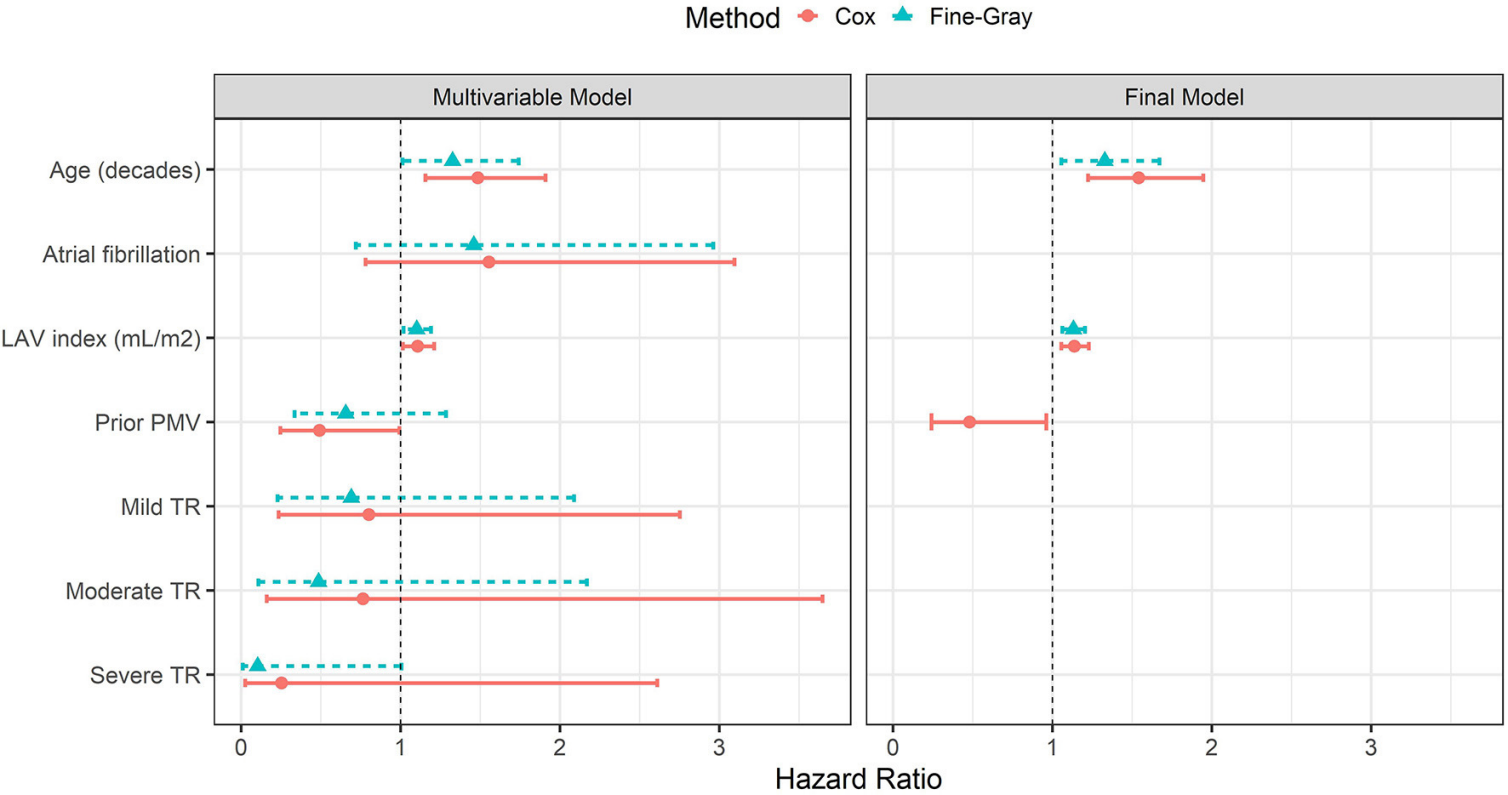
Multivariable predictive models for prediction of mitral regurgitation (MR) progression in patients with rheumatic heart disease (RHD). Cox proportional hazards model considering mitral regurgitation (MR) progression as the primary event and the Fine-Gray model analyzing death and mitral valve replacement as a competing event.

As death and MV replacement constitute a competing risk that may preclude the natural progression of MR, time-to-event analyses were performed considering competing risks. In the Cox proportional hazard regression model, MR progression was the primary outcome, and patients who underwent MV replacement or died were censored. In the Fine-Gray model, MV replacement and death were analyzed as competing events (Table 4). In the multivariable models, age and LA volume were independent predictors of MR progression during the follow-up (Tables 3, 4). In the model with LA volume, AF was no longer a predictor of MR progression. The severity of TR regurgitation was included in the model as this entity is also associated with AF and right atrial dilation. For all variables included, the direction of the association was similar for the rate (Cox model) and incidence (Fine-Gray model) of MR progression. The hazard ratios of each predictor comparing Cox and Fine-Gray models are shown in Figure 3.

**Table 4:** Clinical and echocardiographic characteristics associated with MR progression in patients with RHD: Fine-Gray model.

**At baseline**	**Unadjusted**		**Multivariable model**		**Final model**	
	**HR (95% CI)**	***P* value**	**HR (95% CI)**	***P* value**	**HR (95% CI)**	***P* value**
Age	1.340 (1.072–1.675)	0.010	1.327 (1.012–1.740)	0.041	1.327 (1.055 - 1.669)	**0.016**
Permanent AF	1.928 (1.138–3.266)	0.015	1.460 (0.720–2.961)	0.290		
LA volume index	1.130 (1.060–1.205)	0.000	1.101 (1.018–1.191)	0.016	1.130 (1.060 - 1.203)	**0.000**
Prior PMV	0.567 (0.291–1.102)	0.094	0.656 (0.335–1.285)	0.220		
Mild TR^*^	0.859 (0.291–2.539)	0.780	0.690 (0.228–2.087)	0.510		
Moderate TR	0.885 (0.233–3.352)	0.860	0.485 (0.109–2.169)	0.340		
Severe TR	0.194 (0.021–1.788)	0.150	0.104 (0.011–1.005)	0.051		

*LA, left atrium; AF, atrial fibrillation; PMV, percutaneous mitral valvuloplasty; TR, tricuspid regurgitation*.

* *Reference category was absence of tricuspid regurgitation*.

*Bold numbers mean a p-value < 0.05%*.

We performed a subgroup analysis stratifying according to the MR grade at the follow-up. To do so, we categorized MR progression in moderate (*n* = 45) and severe (*n* = 9), irrespective of the MR grade at baseline. By considering only severe MR in the Cox model, age was the most important predictor of progression (hazard ratio [HR] 2.592; 95% 1.357–4.952), and the effect of LA volume was attenuated (HR 1.121; 95% CI 0.807–1.557). However, the small number of patients in the severe MR category limits this analysis.

**Figure 4:**
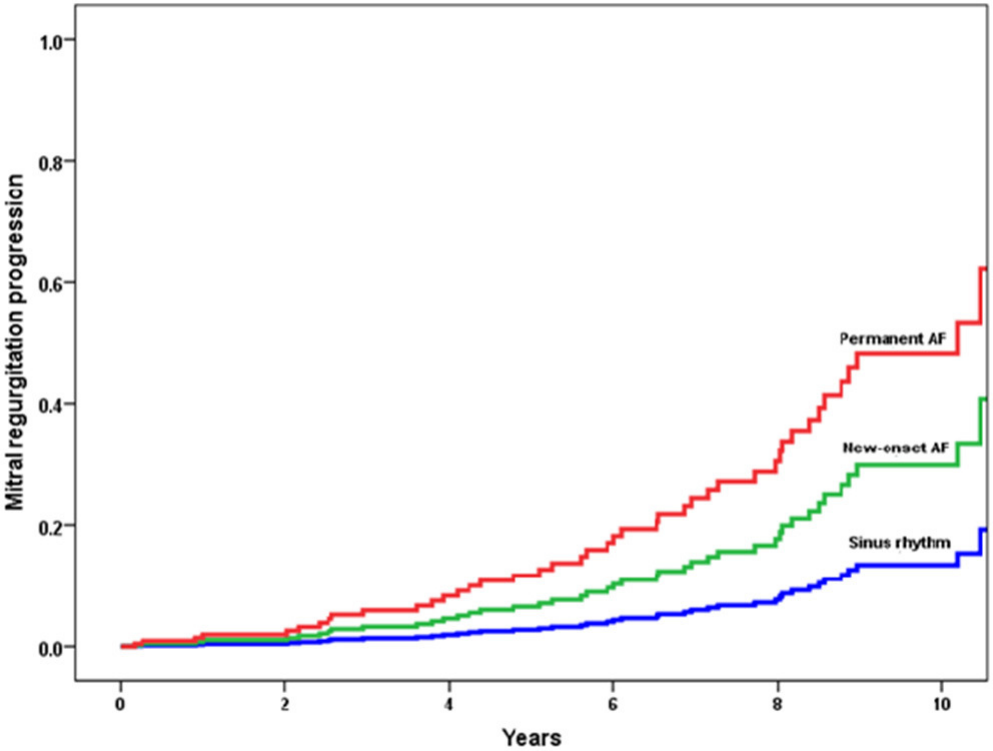
Incidence of mitral regurgitation (MR) progression according to cardiac rhythm. Patients who had sinus rhythm at baseline but with a new-onset of atrial fibrillation during the follow-up were at risk for progression with a hazard ratio of 2.447 (95% CI 1.035–5.788). Patients with permanent atrial fibrillation were at the highest risk for progression with a hazard ratio of 4.459 (95% CI 2.148–9.631) when compared with patients in sinus rhythm.

In the subset of patients in sinus rhythm, 59 patients showed an onset of AF during the course of the follow-up, which was associated with MR progression (HR 2.682; 95% CI 1.133–6.350). Of note, the risk of MR progression was higher in patients with permanent AF at enrollment (HR 4.549; 95% CI 2.148–9.631) compared with those who had new-onset of AF during the follow-up (HR 2.447; 95% CI 1.035–5.788; Figure 4). As expected, patients with new-onset of AF displayed larger LA volume compared with the patients who remained in sinus rhythm (61 and 54 ml/m^2^, respectively).

## Discussion

The natural history of MR varies according to the time course of RHD. While MR in acute carditis may resolve with the control of inflammatory changes, MR at the late disease stages tends to progress over time, which in turn leads to clinical complications (25, 26). The present study showed that in RHD, progression of MR occurs over time with the overall incidence of 2.4 events of progression per 100 patient-years. Age and LA enlargement were major independent determinants of the progression of MR. New-onset AF during the course of the follow-up was associated with MR progression. The study accounts for competing risks to conduct time-to-event analyses appropriately of MR incidence in RHD patients with mixed MV disease.

### Primary MR Progression

The severity of primary MR may increase over time as a consequence of the adverse remodeling of the left atrium and ventricle (27). The degree of regurgitation is an essential determinant of the hemodynamic changes, remodeling of left-sided chambers, and poor outcome. A previous study that includes primary MR, mainly valve prolapse, showed that progression of MR is variable and determined by the progression of lesions or mitral annulus size (28). The most important determinant of marked aggravation of MR is the occurrence of a new flail leaflet followed by an increase in annular diameter, which results in reduced leaflet coaptation. Another study evaluating patients with MV prolapse demonstrated that only mitral annular diameter is a predictor of progression to severe MR (29).

Data on MR progression in patients with RHD are scarce and limited to acute carditis or MR related to percutaneous valve intervention (8, 16–18, 20, 25). In the setting of RHD, given the presence of mixed MV disease, mitral annular enlargement may be induced by both left ventricular and atrial enlargement, which contribute to aggravate the MR severity over time. However, as we included only non-severe MR, the impact of volume overload on the adverse remodeling of the left ventricle might be lower than in severe MR. Additionally, combined valve disease often occurs in RHD and patients may undergo valve replacement for stenosis as the predominant lesion, which influences the natural history of MR progression in the native valve. To address this issue, MV replacement was considered a competing risk that may preclude the occurrence of progression, avoiding biased estimates of progression risk with traditional time-to-event methods (30). The Fine-Gray model constitutes a tool that determines a sub-distribution in a correct way of the role of risk factors, thus taking into account the competition between pairs of events (31).

The significant regurgitant lesion in rheumatic MV has long been considered merely an anatomic variant of its stenotic counterpart, in which retraction of scarred valve leaflets has disrupted the integrity of the mitral seal (8, 32, 33). As the chronic rheumatic process is usually accompanied by at least some fusion of mitral commissures, the relative prevalence of pure regurgitation among hemodynamically severe MV lesions has consistently been reported to be low (8, 34). In agreement with the literature, our population with mixed MV disease, characteristics of pure regurgitation, and pure stenosis were overlapped, which makes it difficult to analyze the progression of the regurgitant lesion alone (13).

### LA Enlargement in MR: The Link Between Primary and Secondary MR

In patients with primary MR, secondary MR can also develop because LA dilation leads to mitral enlargement of the MV annulus. In this context, the overlapping of secondary MR may contribute to further overall progression of the regurgitation. Indeed, there are instances in which both primary MR and secondary MR are present (13).

Left atrial enlargement in MR has been reported either as a compensatory mechanism with the aim to reduce atrial and pulmonary pressure or, conversely, as a marker of poor prognosis (35). Atrial enlargement is accompanied by chronic inflammatory changes, cellular hypertrophy, and wall fibrosis, which leads to reduced compliance and increased LA pressure and risk of AF (36). This association supports the poor prognosis of patients with LA enlargement due to primary MR (36–38).

Although the value of LA enlargement in predicting heart failure and death in the general population has been reported, in primary MR, there are limited data on its prognostic implications (35). A multicenter study showed that LA diameter is an independent predictor of survival in patients with chronic MR due to flail leaflets in sinus rhythm under medical treatment. The association between LA diameter ≥55 mm and the outcome is independent of symptoms or left ventricular dysfunction (37). Another study included 305 patients with MV prolapse and sinus rhythm who underwent MV repair. After a mean follow-up period of 8 years, patients with an area of >30 cm^2^ presented a 2-fold increase in the risk of mortality when compared with those with an area of <25 cm^2^. LA enlargement was a predictor of long-term mortality after surgery for valve repair in sinus rhythm patients (39).

In patients with rheumatic MV disease, a chronic pressure-volume overload on the left atrium leads to a range of adaptive processes that include LA remodeling (40), which encompasses changes in atrial size, function, and shape. LA enlargement also reflects the intrinsic compliance of the left atrium, risk of subsequent AF, and overall disease severity. In the presence of mixed MV disease, LA is affected by both stenosis and regurgitation, which aggravates its remodeling over time with the progression of MR as a consequence of the mitral annulus size. Subsequent progression of primary rheumatic lesions should also be considered. Turbulent flow drives valvular tissue injury, continuously stimulating inflammatory processes and mechanical trauma, which contribute to perpetuate the valvular damage (8). Additionally, patients with RHD often have associated AF, which may contribute to the progression of LA and annular dilation thus increasing the severity of MR. Indeed, there are cumulative pieces of evidence using three-dimensional (3D) echocardiography showing that significant secondary MR can sometimes occurs in AF patients with dilatation of mitral annulus and left atrium. In the present study, 32% of the patients had permanent AF at enrollment and 11% developed AF during the follow-up. Regardless of the cardiac rhythm, LA enlargement was an important predictor of MR progression.

### Study Limitation

Despite providing relevant clinical information on LA remodeling and MV involvement in RHD, this study has some limitations. First, 3D analysis of MV accurately assesses morphology and regurgitation mechanisms. Leaflet remodeling, rather than crude annular dilatation, is associated with the severity of functional MR in patients with AF (41). In our study, mitral annulus by 3D was not assessed and LA dilation was considered a surrogate for mitral annulus enlargement. However, the previous study with 3D-transesophageal echocardiography showed that LA volume is the main predictor of mitral annulus enlargement (42). Moreover, large patient population is required to determine MR progression and 3D analysis of MV in all patients is a challenge. Second, assessment of LA function using novel parameters that include LA strain may be able to detect the onset of decreasing LA compliance and contractile dysfunction that is known to occur in more advanced diseases. In our study, LA function was not assessed. Indeed, atrial disease and remodeling form the basis of the atrial cardiopathy, which plays a critical role in the pathogenesis of AF (43). Third, LV volume and pressure were not measured directly in our study, which influence the amount of MR for a given lesion under different hemodynamic conditions (44).

Finally, the majority of our patients was progressed to moderate MR, which may not have an impact on clinical outcomes. However, the complex nature of mixed MV disease in the setting of RHD makes it necessary to consider all available data to reach a final management decision (13).

## Conclusions

In patients with RHD with a full spectrum of MR severity, progression of MR occurs over time predicted by age and LA volume, corrected by competing risks. LA enlargement may play a role in the link between primary MR and secondary MR in patients with RHD. Assessment of MR progression may provide important insight into the long-term consequences of the disease and the rationale for patient management.

## Data Availability Statement

The raw data supporting the conclusions of this article will be made available by the authors, without undue reservation.

## Ethics Statement

The studies involving human participants were reviewed and approved by UFMG Institutional Ethics Committee (No. 3.586.751). The patients/participants provided their written informed consent to participate in this study.

## Author Contributions

NG, VS, and MN: conception and design of the research. WE, MC, LP, and AP: acquisition of data. JS and MN: analysis and interpretation of data and statistical analysis. RL and WD: obtaining financing. NG and MN: writing of the manuscript and responsible for the overall content as guarantors. RL, JD-B, EA, TT, and JH: critical revision of the manuscript for intellectual content. All authors contributed to the article and approved the submitted version.

## Funding

This study was partly funded by grants from CAPES, FAPEMIG, and CNPq (Project number 431138/2018-4), Brazil. MN and WD are CNPq scholarship recipients. EA and RL labs are funded by the National Institutes of Health grant R01 HL 141917.

## Conflict of Interest

The authors declare that the research was conducted in the absence of any commercial or financial relationships that could be construed as a potential conflict of interest.

## Publisher's Note

All claims expressed in this article are solely those of the authors and do not necessarily represent those of their affiliated organizations, or those of the publisher, the editors and the reviewers. Any product that may be evaluated in this article, or claim that may be made by its manufacturer, is not guaranteed or endorsed by the publisher.
